# On campus dormitories as viral transmission sinks: Phylodynamic insights into student housing networks during the COVID-19 pandemic

**DOI:** 10.1371/journal.ppat.1013666

**Published:** 2025-11-03

**Authors:** Juan Bolanos, Alex Dornburg, April Harris, Samuel Kunkleman, Jannatul Ferdous, William Taylor, Jessica Schlueter, Cynthia Gibas

**Affiliations:** Department of Bioinformatics and Genomics, University of North Carolina at Charlotte, Charlotte, North Carolina, United States of America; American University of Iraq Baghdad, IRAQ

## Abstract

University student housing environments are often viewed as hotspots for infectious disease transmission due to their high-density living conditions and high frequency of interpersonal interactions. During the COVID-19 pandemic, concerns arose that on campus dormitories could serve as amplifiers of viral spread, seeding outbreaks into surrounding off campus student residences. However, whether on campus housing acts as a primary driver of transmission or as a recipient of infections introduced from the broader off campus community remains unresolved. Here, we analyzed 1,431 SARS-CoV-2 genomes collected from students residing on and off campus at the University of North Carolina at Charlotte (UNCC) between September 2020 and May 2022. Sequencing was conducted using an amplicon based whole genome sequencing approach on the Oxford Nanopore PromethION platform. Using Bayesian phylodynamic and ancestral state reconstruction approaches, we traced viral transmission pathways to determine the directionality of spread between residential settings. Our results indicate that transmission from off campus housing consistently seeded on campus dormitory outbreaks. In contrast, viral movement from on campus to off campus housing was minimal. These patterns persisted across all major pandemic waves, regardless of shifting mitigation strategies, and suggest that on campus residences acted as transmission sinks rather than sources of broader student outbreaks. These findings raise the possibility that on campus residences may be more vulnerable than often considered, functioning as epidemiological ‘islands’ that primarily receive infections from off campus sources.

## Author summary

University dormitories are often considered high-risk environments for disease spread due to their crowded living conditions and the inter-connectivity of individuals. This risk has led to the perception that university student housing could act as a major driver of outbreaks, raising concerns that on campus dormitories fuel transmission into surrounding off campus residences and the broader community. However, the actual transmission dynamics between on and off campus communities are often unclear. To address this, we analyzed over 1,400 SARS-CoV-2 genomes from students living on and off campus at the University of North Carolina at Charlotte between September 2020 and May 2022. Using Bayesian phylodynamic and ancestral state reconstruction approaches, we mapped viral transmission pathways and found that infections consistently flowed from off campus residences into on campus dormitories—not the other way around. This pattern persisted across multiple waves of the pandemic, regardless of changing mitigation strategies such as masking, testing, and vaccination policies. Our findings suggest on campus housing functioned more like transmission “islands”—receiving infections from off campus networks but rarely exporting them. These results suggest that universities should shift their focus toward managing transmission in off campus student communities rather than concentrating mitigation efforts solely on dormitory settings. By reframing on campus housing as a transmission *sink* rather than a *source*, our study provides critical insights for improving future outbreak and pandemic response strategies in university environments.

## Introduction

The COVID-19 pandemic has underscored the critical importance of understanding viral transmission dynamics in densely populated environments. University campuses, which serve as microcosms of broader societal interactions, provide a unique lens for studying the spread and evolution of infectious diseases [1]. These environments are characterized by high mobility [2], tightly connected contact networks [3], and varied mitigation strategies in the face of disease outbreaks [4], making them ideal models for understanding how interventions shape transmission within and between residential settings. However, the relationship between on campus and off campus transmission is complex and context-dependent. The potential for campuses to exacerbate transmission became a prevailing concern early in the pandemic, particularly in cities with a high proportion of student residents [5,6].

These concerns were echoed in both public discourse [7–9] and early modeling studies [10]. Some studies suggested that reopening campuses can significantly elevate transmission rates in surrounding communities [11,12] with estimates indicating potential increases of over 50% [13]. Such fears were further amplified by the demographic characteristics of university populations, as younger adults tend to have more frequent social interactions and are more likely to exhibit asymptomatic infections, potentially facilitating undetected viral spread [14,15]. Empirical evidence for sustained transmission from campuses into surrounding communities remains limited. At the same time, robust on campus interventions such as testing, contact tracing, and wastewater surveillance have demonstrated success in containing spread within residential settings [16–18]. The mismatch between the dominant public narrative of campuses as superspreaders and successful mitigation efforts highlights the need for nuanced, data-driven analyses of campus-community transmission patterns to inform context-specific policies to optimize public health outcomes.

Mathematical models and empirical datasets analyzing COVID-19 transmission have identified University campuses as possible superspreaders [19]. These findings are supported by additional studies demonstrating that university-related gatherings and close-contact environments played a significant role in local outbreaks during peak transmission periods [20]. Evidence from structured educational settings further indicates that large infection clusters can rapidly emerge from individual events, reinforcing the importance of social context in shaping transmission dynamics [21]. However, the extent to which on campus dormitories contribute to spread across dispersed off campus university residential settings is not clear. The dynamics of dormitory populations may differ from the dynamics of the university population as a whole. For example, some universities implemented intervention strategies that significantly lowered transmission within campus housing communities [22]. These outcomes align with mathematical simulations that suggest robust contact tracing and demonstrate that intervention programs can successfully mitigate outbreaks in university settings [23]. College campuses also vary dramatically in their demographics and geographical settings, both of which are highly correlated to incidence patterns [24–26]. University residential settings span the full urban to rural gradient, from campuses located within major population centers to those located within small isolated communities. Most studies have focused on isolated extremes of this spectrum of settings [20], raising the question of how universities in urban environments with off campus residences scattered throughout high density metropolitan housing areas differ in transmission risks and dynamics.

Urban campuses are characterized by high population densities, extensive student clustering, and substantial daily movement between on campus dormitories, off campus apartments, and other communal spaces [27,28]. The high frequency of interpersonal interactions, shared spaces, and proximity to common gathering areas could position on campus housing as viral incidence amplification sites that facilitate streams of transmission to off campus residences. An alternative hypothesis, though, is that on campus residences may act as epidemiological ‘islands’, where infections are introduced by contact with individuals residing off campus who have had greater exposure to contacts in the broader community. In this scenario,viral strains are introduced to campus, and may circulate within the campus population, but contribute little to overall community spread beyond the campus [16,29]. Supporting this latter scenario, Purdue University detected at least 10 independent introductions of the gamma variant onto its campus in 2021 [30], emphasizing the possibility of multiple entry points into on campus residences. Disentangling these contrasting hypotheses, akin to classic source-sink dynamics in biogeography [31–34], is critical for understanding the degree to which student housing acts as a self-contained transmission network or as a gateway for broader spillover into the university’s residential community. To address this question, we apply a methodological approach capable of tracing viral movement with high resolution. Traditional epidemiological metrics such as incidence data have been widely used to reveal overall trends in infection rates between campus and other environments [11]. However, these metrics cannot resolve the history of transmission between individuals in the absence of detailed contact tracing. Recent advances in phylodynamic approaches facilitate the integration of viral genomic data with epidemiological models, which permit reconstruction of transmission pathways with robust resolution [35,36]. Such approaches have been widely used to track the spread of infectious diseases at global and national scales [37–39]. Instances of their application to university campuses is less common [20], creating a knowledge gap in our understanding of general trends in the transmission dynamics of viruses between campus populations.

**Fig 1:**
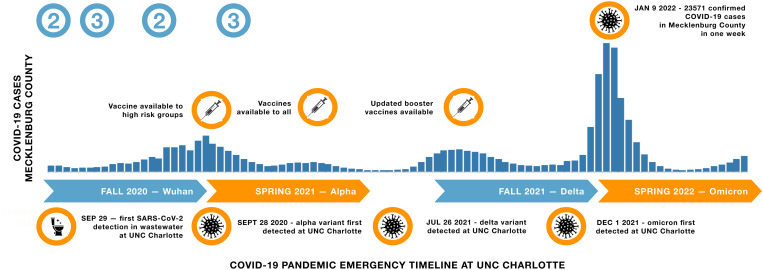
Timeline of major COVID-19 pandemic events at UNC Charlotte and in Mecklenburg County, NC, relative to NC-DHHS case incidence data for the study period (August 15, 2020 to May 15, 2022). We divided the timeline into four phases aligned to university semesters. The later time periods roughly correspond to the timing of introduction to North Carolina of the dominant variants of concern (VOCs) that emerged during winter 2020 (alpha), summer 2021 (delta) and winter 2021 (omicron) and are named accordingly. Reported COVID-19 incidence is depicted by the cool bar graph over the course of the study. Vaccination rollouts and other events are indicated by warm circles at their corresponding time points. Cool circles labelled (2) or (3) correspond to the emergency conditions designated by the state. North Carolina went into full lock down–Phase 1 conditions– at the onset of the COVID-19 pandemic. However, during the period of this study the state of North Carolina had lifted phase 1 conditions, remaining primarily in Phase 2 or Phase 3 of emergency guidelines. UNC Charlotte mirrored these guidelines. The campus remained open during the entire period, managing exposure via density reduction between August 2020 and May 2021, and returning to full occupancy for the second year of the pandemic declaration. The University’s actions in the first year closely paralleled the statewide phases. In the second year, the university delayed the start of in-person instruction each semester by 2-3 weeks, due to the delta and omicron waves. During the entire 2-year period, wastewater surveillance of dormitories triggered testing of on campus residents, and additional re-entry, symptomatic, case contact, and random surveillance testing of the student population continued during that time (see supplemental materials for additional details).

Here we integrated extensive SARS-CoV-2 genomic sequence data (n = 1431 genomes) with public health and intervention data from the University of North Carolina at Charlotte (UNCC), a mid-sized urban institution serving over 31,000 students within the densely populated Charlotte metropolitan region [40]. We included sequences collected between September 2020 and May 2022. This period encompassed waves of alpha, delta and omicron viral variants, as well as the introduction of vaccines. During this time, the university maintained active mitigation programs including testing, wastewater surveillance, masking, distancing, and case isolation (Fig 1).

We analyzed these genomic and epidemiological data in a Bayesian phylodynamic framework. We apply time-calibrated phylogenies and discrete ancestral state reconstructions [41] to test three key hypotheses regarding the role of student housing settings in transmission dynamics. First, we assessed the degree to which transmission dynamics between on- and off campus populations reflect alternate possible source-sink scenarios. Specifically, we determined whether the university primarily drives spillover into surrounding off campus housing or functions as a recipient of infections introduced from off campus residences. Second, we evaluated whether shifts in on campus mitigation strategies, such as masking mandates, asymptomatic and symptomatic testing, and dormitory occupancy limits, corresponded to measurable changes in transmission patterns within and between campus and off campus residences. Lastly, we examined whether viral lineage dynamics in residences represented early indicators of broader outbreaks or if infections lagged behind those in the greater Charlotte metropolitan area. Collectively, our results challenge the prevailing popular media notion that on campus residences act as primary superspreader hubs or reservoirs amplifying off campus transmission. Instead, they show a higher rate of transmission into the campus under all mitigation conditions examined.

## Results & discussion

### Campuses dormitories can act as sinks of viral transmission

**Fig 2:**
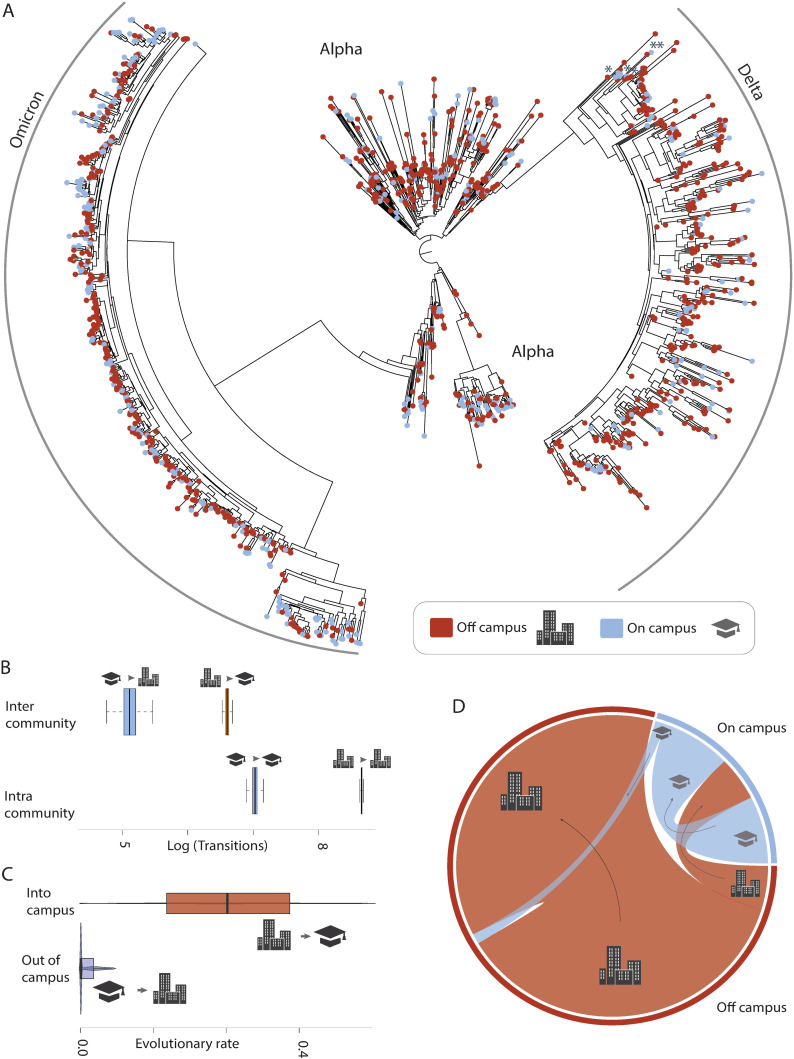
Transition history and transition dynamics between on and off campus communities during the study period. (A) Maximum likelihood tree topology of SARS-CoV-2 sequences collected during this study. Tip labels indicate whether samples were derived from on campus (cool shading) or off campus (warm shading) locations. * symbols in the delta strain portion of the phylogeny demarcate lineages identified as alpha by diagnostic mutations, but are phylogenetically resolved within delta (see supplemental materials). (B) Box plots depict the estimated quantiles of the log-transformed number of transitions occurring between communities. Top row reflects the frequency of transmissions occurring between the campus and off campus community (left) or between the off campus community and the campus (right). Bottom row reflects the frequency of transmissions within the campus (left) or within the off campus community (right). Cartoons above each box plot indicate the transmission mode and correspond to the legend in (A). (C) The tempo of transmission between communities. Violin plots depict the estimated distribution of evolutionary transition rates estimated in BEAST, truncated to the 3rd quartile (75%). (D) Relative frequency of transmissions between and within communities. Chord diagram depicts the relative frequency of transmissions, shaded by community as in (A). Outer bands correspond to total relative cases for each community, and inner chords illustrate transmission mode scaled to their frequency. Arrows and cartoons illustrate the direction of transmission.

We sequenced viral genomes from 1444 SARS-CoV-2 positive clinical samples collected at the University of North Carolina at Charlotte (UNCC) between September, 2020 and May, 2022, of which 13 did not pass quality control and were excluded from downstream analysis. The samples sequenced were from 470 and 961 patients living on campus or off campus, respectively. Using this data, we estimated a time calibrated Bayesian phylogenetic framework to model the transmission pathways of SARS-CoV-2 between on campus and off campus student populations via ancestral state reconstruction (ASR). (**Fig 2A**). Given the location of the school and the large number of commuter students, our initial hypothesis was that on campus dormitories could act as a primary source of infections, fueling outbreaks among off campus students and facilitating spread into the broader community. However, our results strongly support that this is not the case (**Fig 2**). Assessing the distribution of off and on campus incidence across the phylogenetic history of transmission revealed numerous shifts between on and off campus residences (**Fig 2A**). In most cases, transmission pathways reflect bursts of infections within either the on or off campus community, reflecting a higher rate of transmission within either community than between communities (**Fig 2B**). In cases of transmission between on and off campus residences, transmission from off campus residences into campus residences dramatically outnumber the reverse (**Fig 2C).** This asymmetry suggests that viral introductions into the university environment were primarily seeded from off campus sources rather than spreading outward into the off campus community, with as many as one fifth of the total number of cases on campus being a direct consequence of transmission from off campus residents. These results were robust to bootstrapping of the data (S1 Fig), and they challenge assumptions that university dormitory populations act as amplifiers of broader transmission. Rather, our findings suggest the on campus student population is a transmission sink within the larger context of the student housing network.

Universities are often assumed to be amplifiers of viral transmission due to high-density living and frequent interpersonal interactions [19]. However, our results suggest that the off campus student housing was the primary driver of infection trends at UNC Charlotte, with the on campus population largely reflecting, rather than shaping, viral transmission dynamics among off campus students. The clustering of on campus infections following introductions from the off campus population indicates that once introduced, transmission among those students living in the university environment was sustained at lower overall rates than among those living in the surrounding community. This pattern suggests that on campus student residences are vulnerable to external seeding events and may not act as primary drivers of viral transmission in the campus community. More broadly, these results suggest that on campus housing may follow similar transmission dynamics as other highly structured sub-populations such as nursing homes or long term care facilities [42]. It is becoming increasingly evident that community-wide mobility networks are predictive of infection pathways in the urban landscape [43], suggesting that the degree of connection of students to the community outside of campus is likely a major determinant of transmission in stratified campus communities.

As viral genomic data from the COVID-19 pandemic continue to be analyzed, evidence has accumulated for multiple introductions into urban college campuses that seeded in situ outbreaks, as well as for limited spillover into surrounding communities [30,44]. It may be tempting to extrapolate our findings to consider spillover from UNCC into the broader metropolitan community. However, doing so exceeds the intended scope and resolution of our study. Like other campus-based genomic surveillance efforts, our dataset lacks the spatial granularity and individual-level mobility data necessary to characterize urban transmission dynamics such as evaluating whether certain city regions disproportionately contributed to off campus infections [45]. To understand the placement of on-campus student housing in the wider community transmission network would require access to metadata that the university did not share with us under the terms of our IRB protocol. Instead, our findings underscore the value of treating university populations as stratified subgroups within larger urban transmission networks [46,47]. Rather than assuming university residences uniformly influence regional trends, our data suggests that integrating data from campus-based healthcare and surveillance systems into models of urban mobility and residential structure [48] could offer nuanced information of high utility for public health. Future work focusing on such an integration will be essential for understanding how structured populations, like university students in this case, mediate pathogen flow across the urban landscape, vital information for designing interventions tailored to the dynamics of interconnected, stratified communities.

### Mitigation strategies impact transmission rates, not source-sink dynamics

To assess whether shifts in mitigation strategies corresponded to changes in transmission dynamics between on campus and off campus student housing, we analyzed SARS-CoV-2 lineage movement across four major phases of the pandemic: Wuhan (Fall 2020), Alpha (Spring–Summer 2021), Delta (Fall 2021), and Omicron (Spring 2022). Throughout this period, mitigation strategies transitioned from stringent controls—such as reducing dormitory occupancy to one-third capacity, enforcing masking and social distancing, and relocating SARS-CoV-2-positive students to designated isolation dormitories—to increasingly relaxed control measures that culminated in the resumption of in-person learning with many previous mitigation strategies optional (**see supplemental materials**). As measures relaxed, so did compliance with self-reported symptom tracking, mirroring broader trends in pandemic fatigue and shifting public health priorities [49–51]. These changes in campus policy largely aligned with evolving state and federal guidelines for North Carolina (**supplemental materials**), and raise the possibility that viral transmission dynamics between off and on campus may experience concomitant shifts that amplify spillover between these communities. However, our analyses strongly suggest this to not be the case.

**Fig 3:**
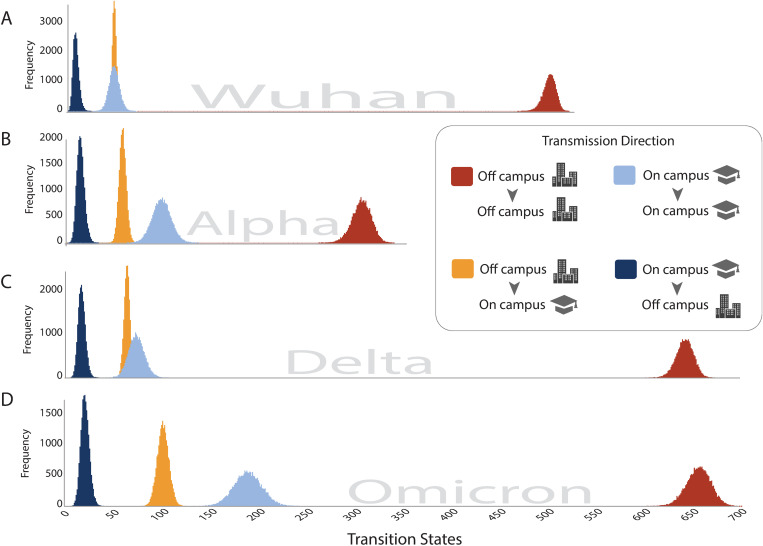
State transition distributions across four major waves of the pandemic: (A) Wuhan; (B) Alpha; (C) Delta; and (D) Omicron. The frequency (Y axis) of transitions (X axis) between and within residence types are indicated by the shadings in the figure legend with areas indicating the direction of transmission for each shading (i.e., off campus to on campus).

Considering transmission dynamics across major variant-driven case surges of the pandemic reveals incidence trends that consistently aligned with mitigation shifts (**Fig 3**). In general, relaxation of restrictions led to higher frequencies of incidence in the on campus population, as well as a separation between the distribution of transitions between on campus cases, and transitions from off campus onto campus, with higher levels of on campus circulation (**Fig 3**). For example, during the initial Wuhan phase, when stringent mitigation measures such as mask mandates, social distancing, and routine testing were in place, on campus transmission was equivalent to transitions from off to on campus (**Fig 3A**). In contrast, incidence levels during Alpha, when many restrictions were lifted, led to on campus transmission events outpacing the transitions from off to on campus (**Fig 3B**). This same pattern was repeated when comparing delta and omicron, the latter of which vastly exceeded transmission levels observed in other phases of the pandemic, both on and off campus (Fig 3C, 3D and S1 Table). These incidence patterns reflect a global trend, as Omicron’s increased transmissibility and immune evasion properties contributed to surges of infection worldwide [52–54].

**Fig 4:**
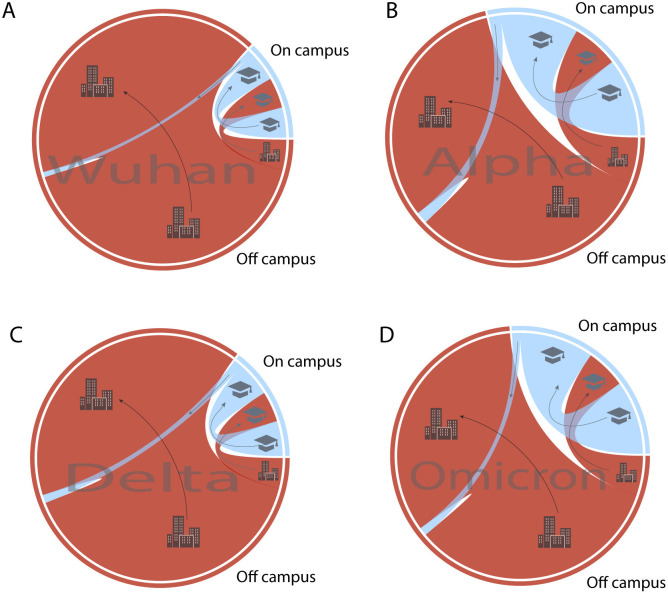
Transmission dynamics between on and off campus across variant waves: (A) Wuhan; (B) Alpha; (C) Delta; and (D) Omicron. The relative frequency of transmissions between and within communities is depicted in chord diagram, shaded by community as in **Fig 2**. Outer bands correspond to total relative cases for each community, and inner chords illustrate transmission mode scaled to their frequency. Arrows and cartoons illustrate the direction of transmission.

However, regardless of time period, movement of viruses from the campus residences into the off campus community remained unchanged. Transmission occurred primarily from the off campus residences into on campus residences, rather than the reverse (**Fig 4**). Even as on campus transmission increased during Omicron, there was no corresponding rise in outward viral movement from campus into the community (**Figs 4D and 4**). These patterns were robust to resampling, indicating this result is not an artifact of differential incidence levels between on campus and off campus populations alone (S1 Fig). Instead, the persistent asymmetry between off campus to on campus and on campus to off campus transitions suggests that the primary drivers of viral introduction into the university were external, rather than originating within campus populations. This pattern mirrors findings from other university settings during Omicron, where campuses exhibited high in situ transmission but limited spillover into the surrounding community [17,55]. These results suggest that, regardless of mitigation measures, UNC Charlotte continued to act primarily as a sink rather than a source of community transmission.

There is no doubt that initial pandemic restrictions and mitigation strategies saved millions of lives across the globe [56]. Similarly, targeted university interventions such as masking mandates and routine testing are estimated to have reduced infections on University campuses by an order of magnitude or more [57]. However, universities, like other sectors [58], had to navigate a complex trade-off between the immediate health benefits of mitigation and the long-term academic, social, and economic consequences of restrictive policies [59,60]. Advances in genomic surveillance methods [61,62] as well as computational models have come to play a crucial role in balancing disease suppression with the need for in-person education [63,64]. Our results suggest that placing the university within the broader framework of urban disease ecology could provide vital additional context to multiple urban sectors. In particular, our finding of a marked asymmetry in viral transmission, that persisted across mitigation strategies, suggests that on campus residences such as those at UNC Charlotte may be epidemiologically insular, acting as relatively self-contained transmission units rather than perpetual community wide spreaders. Further work leveraging computational [65] and phylodynamic approaches [35,66] are critically needed to assess whether similar trends hold across other urban campuses.

### On campus infection waves lag behind off campus waves

**Fig 5:**
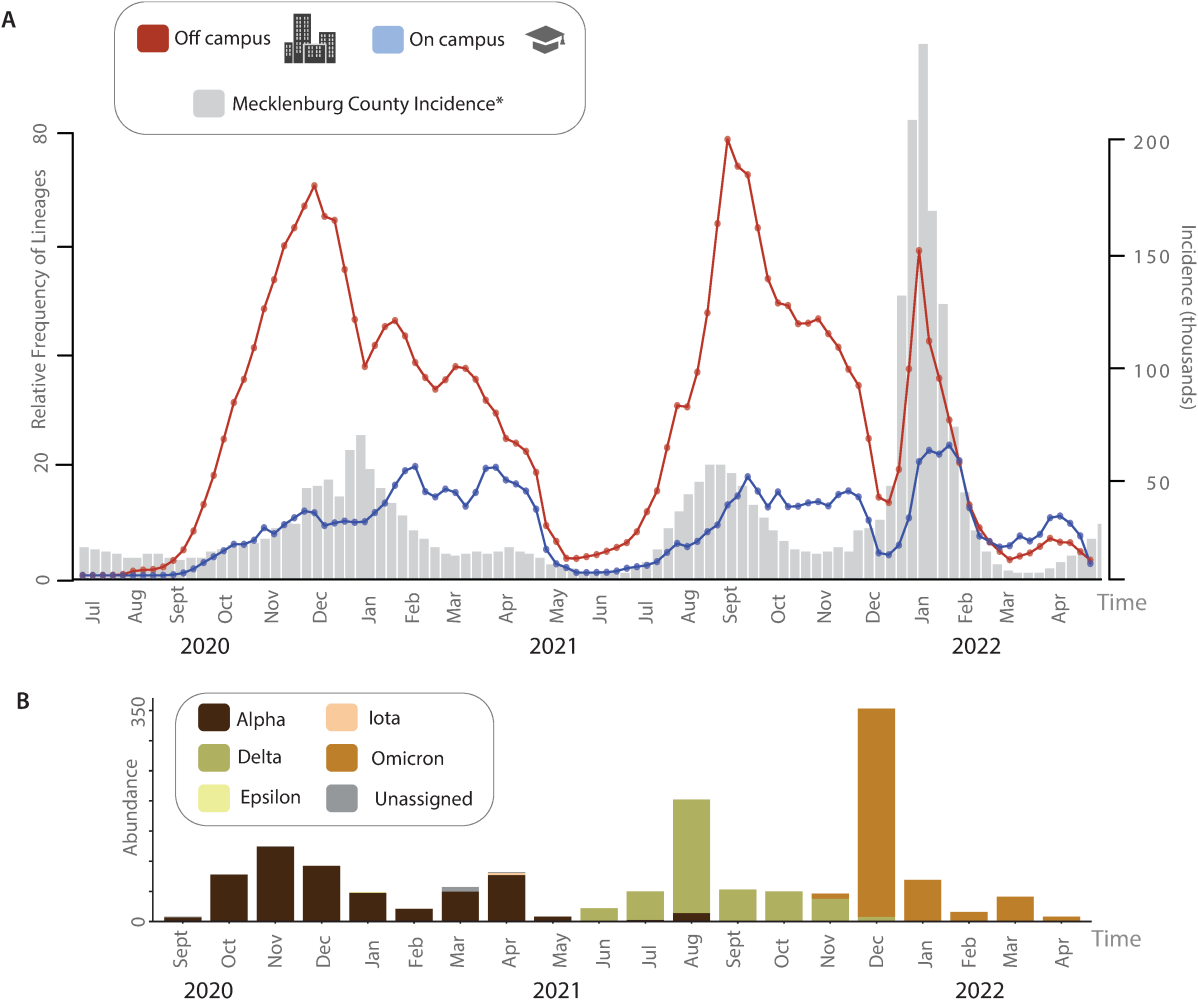
Trends in lineages through time and incidence. (A) SARS-COV-2 relative frequency of lineages through time for off campus (warm shading) and on campus (cool shading) residences plotted in comparison to incidence in Mecklenburg county (gray). Circles in lineage through time plots represent specific time points. (B). Abundance of circulating strains during the time period depicted in A. Strains are indicated in the legend.

If on campus student housing were a major source of viral transmission, we would expect infection waves among dormitory residents to precede or at least coincide with those in off campus housing. However, our phylodynamic analyses reveal a consistent lag in on campus infection waves relative to those in off campus housing, with peaks in on campus lineage counts occurring after surges in off campus populations (**Fig 5A**). This pattern is particularly pronounced for the Delta and Omicron waves (**Fig 5**), where variant-driven outbreaks first became established in off campus housing before spreading into dormitories. Incidence peaks largely align with the broader emergence of new variants of concern in the surrounding community (**Fig 5B**), and the delayed pattern of spread is consistent with the expected transmission lag when considering the average incubation period of SARS-CoV-2, which ranges from 5 to 14 days [67,68]. The variation between off and on campus incidence patterns over time was particularly pronounced during periods of higher mitigation on campus, which resulted in an overall muting of peak incidence patterns (S2 Fig), and likely reflects the efficacy of mitigation measures. Collectively, these results imply that consistent transmission from source off campus residences seeded numerous on campus cases and outbreaks during all waves of the pandemic, with times of heightened use of on campus mitigation strategies, controlled environments, and testing preventing further in situ outbreaks.

Throughout the COVID-19 pandemic, transmission dynamics often varied spatially, with some areas experiencing infection waves weeks ahead of others [69]. This staggered pattern of viral spread can occur both at larger geographic scales (e.g., hemisphere, country, etc) as well as at more localized scales (e.g., state, county, etc) [70,71]. Understanding variation in the spread of disease is of particular concern in densely populated urban environments where social connectivity, mobility patterns, and mitigation strategies influence the timing and intensity of outbreaks [72]. If university on campus residences were major sources of community spread, we would expect on campus surges to precede infection waves in off campus residences, that, in turn, would likely then seed transmission through the larger metropolitan area. However, our findings strongly suggest the reverse. The on campus residences at UNC Charlotte largely acted as recipients of the off campus community viral spread, rather than an independent epicenter of transmission. Moreover, the on campus community infection waves lagged behind the dominant incidence waves recorded for the region (**Fig 5A**). This pattern of transmission raises the possibility that off campus housing acts as a gateway enabling viruses circulating in the community to enter and circulate on campus. Whether there are asymmetrical pathways of transmission such as this between the broader community and the campus remains unknown and represents an exciting area of future research.

## Conclusion

Our findings demonstrate that on campus student housing at UNC Charlotte functioned as a transmission sink rather than a source of campus community-wide viral spread throughout the COVID-19 pandemic. By leveraging whole-genome sequencing and Bayesian phylogenetic analyses, we reconstructed SARS-CoV-2 transmission pathways and found strong evidence that viral introductions into dormitories overwhelmingly originated from off campus housing rather than spreading outward from campus into the broader student population. This pattern persisted across multiple pandemic waves and mitigation phases, suggesting a consistent epidemiological role for on campus residences as recipients of community transmission rather than amplifiers of broader outbreaks. Importantly, even during periods of high incidence—such as the Omicron surge—there was no corresponding increase in outward viral movement from dormitories to off campus residences. These results suggest on campus housing to possibly be similar in expected viral transmission dynamics to other structured, high-density subpopulations that have limited interactions with the broader community, such as long-term care or military facilities, in which external introductions predominantly seed internal outbreaks [73–75]. Future work assessing additional universities with similar on and off campus residence structures is critically needed to determine the degree to which these transmission source-sink dynamics persist across institutional and geographic contexts.

## Materials and methods

### Ethics statement

This project was approved by UNC Charlotte’s Institutional Review Board under protocol IRBIS-21–0113. Viral sequencing was permitted by this protocol as a secondary use of discarded clinical test specimens and did not require a consent process. Specimens were anonymized, retaining only date, positivity status of the COVID-19 test, and building-level residence location where the positive test was collected. Sequences mapping to the human genome were filtered out of all datasets prior to deposition in GISAID and NCBI.

### Sample collection and RNA extraction

Samples were obtained from the Covid Testing Center (later absorbed into the Student Health Center) at the University of North Carolina at Charlotte. Collection involved buccal nasal swabs from symptomatic as well as asymptomatic students and employees as part of routine surveillance and diagnostic testing. Testing was not contingent on SARS-CoV-2 infection status at the time of sampling. Viral RNA extraction was performed by the respective facility. StarMed utilized the Kingfisher system with the MagMAX-96 Total RNA Isolation Kit (Catalog No: AM1830) for RNA extraction, while the UNC Charlotte testing center employed the KingFisher Flex with the MagMAX Viral/Pathogen II (MVP II) Nucleic Acid Isolation Kit (Catalog No: A48383). After extraction, qPCR was conducted to detect SARS-CoV-2 positive samples. UNC Charlotte used three probes (N1, N2, and S primers, designed by the CDC) with the TaqPath COVID-19 Combo Kit (Catalog No: A47814), whereas StarMed relied solely on the N1 primer for detection, using the same kit. Only RNA from SARS-CoV-2 positive samples was forwarded for sequencing. This sampling resulted in 1444 unique positive cases of which 1431 were successfully sequenced. Samples were excluded if sequencing failed, if associated metadata (e.g., housing status) was incomplete, or if sequences did not meet quality thresholds based on Pangolin pass/fail status and Nextclade assessment. This sample represents nearly 90% of all known campus associated SARS-CoV-2 cases during the study period, with only approximately 200 additional cases voluntarily reported by individuals who were tested in locations away from campus without a surveillance sequencing program.

### Library preparation and sequencing

RNA extracts of clinical samples were reverse-transcribed and amplified in PCR according to a tiling amplicon protocol modified from Pater et al. [76]. A fully detailed guide to this modified protocol is available on protocols.io [77]. Briefly, first we transformed extracted RNA into complementary DNA (cDNA), with cDNA enrichment of the SARS-CoV-2 genome performed by multiplexing PCR with two pools of ARTIC primers. For each sample, the ARTIC PCR products were pooled and amplicons were isolated through magnetic Solid Phase Reversible Immobilization (SPRI) beads and quantified by Qubit fluorescence. Subsequent sequencing library preparation comprised amplicon end-prep, sample barcoding, sample pooling and cleaning, and sequencing adapter ligation. Finally, the sample library was loaded onto an R9 flow cell (Oxford Nanopore Technologies, ONT) and sequenced using the ONT PromethION instrument. Manufacturer-recommended settings for the LSK-109 genomic sequencing by ligation kit, Native Barcode Expansion Kit (Exp-NBD196), and FLO-PRO002 flow cell were applied at the start of the run.

### Sequence assembly and variant calling

Basecalling was initially carried out in real time using the Oxford Nanopore guppy software with high accuracy setting, via the MinKnow user interface on the ONT PromethION instrument. During spring of 2021, updates to ONT basecalling algorithms became available and were applied as they were released. In order to avoid biases arising due to changes in basecalling algorithms, sequences were re-basecalled from raw fast5 files using the ONT 1.2.1 “super high accuracy” basecalling method. Assembly and variant calling of SARS-CoV-2 sequences was performed using a modified version of the ARTIC ‘fieldbioinformatics’ pipeline (https://github.com/artic-network/fieldbioinformatics). Briefly, reads were filtered and trimmed with guppyplex to remove V3 adapter sequences and to exclude chimeric sequences smaller than 250 bases and longer than 700 bases. Alignments were constructed using minimap2, followed by medaka (https://github.com/nanoporetech/medaka) for polishing, variant calling, and consensus building using the ‘r941_prom_high_g360’ model.

Consensus sequences were classified using Pangolin (https://github.com/cov-lineages/pangolin) versions 3.1.11, 3.1.14, 3.1.16, 3.1.17, 3.1.19, 3.1.20, and 4.1.2 to ensure accurate lineage assignments over the course of the study (For example, earlier versions would not be able to accurately classify Delta or Omicron due to their unique mutation signature). Sequences were inspected for quality based on the pass/fail metrics in Pangolin that assess minimum sequence length and maximum allowable ambiguous bases (N content). Of the sequenceable samples, 13 failed quality control at this classification step and were excluded from downstream analyses. Additional quality control was conducted using Nextclade [78], which assesses missing data, ambiguous sites, mutation clusters, stop codons, and frame shifts for SARS-CoV-2 (https://github.com/nextstrain/ncov).

### Phylogenetic analyses

A total of 1444 viral samples were collected from both on campus and off campus populations between September, 2020 and May, 2022. Of these, 1431 passed the sequence quality control assessments described above. All sequences included in the phylogenetic analysis passed stringent quality control filters based on Pangolin and Nextclade assessments, ensuring that only high confidence sequences were used, thereby mitigating alignment artifacts. The final set of sequences (available in GISAID with EpiSet ID: EPI_SET_250220my) is composed of 470 samples originating from on campus students and 961 from off campus individuals. We used the Wuhan strain (accession NC_045512) to serve as a reference genome and outgroup for analysis. Sequences were aligned using MAFFT v7.525 [79]. To estimate the evolutionary history of our collected samples, we used MAPLE (MAximum Parsimonious Likelihood Likelihood Estimation), a computational tool designed for large-scale phylogenetic inference of epidemiological datasets that has been shown to significantly reduce computational analysis time while increasing accuracy relative to other likelihood approaches [80].

We used RelTime with Dated Tips (RTDT) [81] in Mega X [82] to time calibrate the MAPLE inferred maximum likelihood topology, allowing us to estimate the transmission timing of SARS-CoV-2 across on campus and off campus student housing. RTDT is an algebraic method designed for estimating divergence times from temporally sampled molecular sequences, making it particularly well-suited for analyzing pandemic-scale datasets with precise sampling time information. RTDT incorporates a relative rate framework, enabling computational efficiency while maintaining a level of accuracy that is par with more computationally complex Bayesian approaches to divergence time estimation such as those implemented in BEAST [83–85], including for the analysis of SARS-CoV-2 data [86]. Divergence times were calibrated using the time each viral genome was sampled.

### Reconstructing the history of transmission

We used the discrete phylogeographic diffusion model implemented in BEAST v2.6.7 [41,87,88] to reconstruct the transmission history of SARS-CoV-2 between student housing communities. This model integrates ancestral state reconstruction (ASR), a well-established method in evolutionary inference [89–92], with a diffusion model that models viral spread through distinct transmission events between predefined geographic units [93]. This approach allowed us to quantify the directionality and frequency of transitions between on campus and off campus housing while integrating branch-specific rate variation to accommodate heterogeneity in transmission dynamics [93]. We fixed the time calibrated phylogeny estimated above, and ran two independent Markov chains for up to 250,000,000 generations to estimate transmission histories. Runs were stopped at 232,979,000 generations as inspection of the likelihood and other parameter values in Tracer v1.7.2 [94] indicated convergence between runs, with all effective sample size (ESS) values above 200, indicating effective sampling of the target distributions. To verify that posterior estimates were informed by the empirical data, we conducted an additional BEAST analysis in which parameters were sampled exclusively from the prior distributions. We used the Babel package (https://github.com/rbouckaert/Babel) to quantify the distribution of on or off campus lineages through time with the LineagesThroughTimeCounter function, and also quantified transitions between on and off campus using the transitionStateCounter function. Resulting transition histories were imported into R visualized using Circos plots with functions from circlize v0.4.16 [95,96] treedataverse v0.0.1 [97,98], and tidyverse v0.4.6 [99] libraries for additional visualizations. Transmission histories were visually compared to incidence frequencies in Mecklenburg County using public incidence data available from the North Carolina Respiratory Virus Summary Dashboard (https://covid19.ncdhhs.gov/dashboard).

Our dataset captured approximately 90% of all cases known within these populations during the study period. However, it is possible that the uneven levels of incidence between off and on campus might bias estimation of transmission dynamics. To assess the degree to which possible sampling biases of campus populations impact estimated transmission dynamics, the above analyses were repeated on randomly subsampled data sets with predefined on campus:off campus ratios (1:5, 2:3, 1:1, 3:2, 5:1). For each set of datasets at a given ratio (e.g., 5 on campus sampler per very 1 off campus sample), total transmissions between and within population (off campus to off campus, on campus to on campus, off campus to on campus, and on campus to off campus) were quantified to ensure a consistent signal of source-sink dynamics relative to that based on the observed incidences of COVID-19.

### Limitations

This study provides high resolution insights into SARS-CoV-2 transmission dynamics within a university population that comprises students living in both on campus dormitories and off campus living arrangements. It is important to note some key limitations. Most notably, this analysis does not incorporate viral genomic data from the wider Charlotte metropolitan community. Therefore our findings cannot be extrapolated to the dynamics between on campus student housing and the broader community, since the absence of wider community sampling constraints our ability to determine whether viral introductions into the university setting were seeded from specific external sources within the city or whether on campus residences contributed transmission back into the surrounding community. Similar limitations have been noted in other university focused genomic studies, where limited community sampling impeded inferences about transmission linkages beyond campus boundaries [45]. Although we included a small number of samples from university employees (categorized as off campus), our dataset is predominantly student focused and may underrepresent other demographic or occupational groups promoting community spread.

In order to fully understand the role of on campus student housing in relation to the wider community transmission dynamics a future study that integrates campus based data with representative community sampling constitutes an important and exciting research direction. Expansion of our dataset to include data from the broader community generated by other groups involved in regional surveillance effort will be the focus of a future study, to delineate the ecological role of urban campuses in pandemic spread and to inform targeted public health interventions across institutional and municipal boundaries.

## Supporting information

S1 Fig Effect of subsampling on estimated transmission dynamics. State transition frequencies were evaluated across phylogenies randomly subsampled at predefined on-campus:off-campus sampling ratios (1:5, 2:3, 1:1, 3:2, 5:1). Transition categories (off-campus to off-campus, on-campus to on-campus, off-campus to on-campus, and on-campus to off-campus) were compared across biased sampling strategies to assess if uneven frequencies of infection between populations would impact the trends observed in the empirical frequencies. Across all sampling strategies, the relative relationship between transition frequencies remained consistent, with only the absolute number of transmission events within the off-campus community modulating. Bars represent the mean number of transitions across each simulation and category, with box plots indicating the 95% confidence interval for each distribution. The x-axis denotes the resampling ratio, and the y-axis indicates the number of inferred transmission events. Off campus and on campus biased resampling results are indicated by shading on the x-axis.
(TIF)

S2 Fig Trends in lineages through time and incidence during the major waves of COVID-19 pandemic that occurred during the study period (A) Wuhan, (B) Alpha, (C) Delta, (D) Omicron. Lines indicate the relative frequency of SARS-COV-2 lineages through time for off-campus (warm shading) and on-campus (cool shading). Circles in lineage through time plots represent specific time points.
(TIF)

S3 Fig Transmission dynamics between on and off campus across variant waves: (A) Wuhan; (B) Alpha; (C) Delta; and (D) Omicron. The relative frequency of transmissions between and within communities is depicted in chord diagram, shaded by community. Outer bands correspond to total relative cases for each community, and inner chords illustrate transmission mode scaled to their frequency. Arrows and cartoons illustrate the direction of transmission. These visualizations complement the data presented in S1 Table and illustrate the consistent asymmetry of transmission from off-campus to on-campus populations across all pandemic phases.
(TIF)

S1 Table Mean number of transmissions estimated within and between populations during major waves of the COVID-19 Pandemic. (DOCX)

S2 Table Number of high quality SARS-CoV-2 genomes sequenced per variant phase. Variant phases were defined based on sampling dates aligned with periods of variant dominance Wuhan (Fall 2020), Alpha (Spring–Summer 2021), Delta (Fall 2021), and Omicron (Spring 2022). Counts reflect genomes passing quality control filters. The Wuhan reference genome used for rooting the phylogeny is excluded from these totals.
(DOCX)

S1 Text Supplementary results. (DOCX)

## References

[1] Stubbs CW, Springer M, Thomas TS. The Impacts of Testing Cadence, Mode of Instruction, and Student Density on Fall 2020 COVID-19 Rates On Campus. Cold Spring Harbor Laboratory. 2020. doi: 10.1101/2020.12.08.20244574

[2] Bhandari R, Blumenthal P. Global Student Mobility and the Twenty-First Century Silk Road: National Trends and New Directions. International Students and Global Mobility in Higher Education. Palgrave Macmillan US. 2011. 1–23. doi: 10.1057/9780230117143_1

[3] Israel U, Koester BP, McKay TA. Campus connections: Student and course networks in higher education. Innov High Educ. 2020;45:135–51.

[4] Klein B, Generous N, Chinazzi M, Bhadricha Z, Gunashekar R, Kori P, et al. Higher education responses to COVID-19 in the United States: Evidence for the impacts of university policy. PLOS Digit Health. 2022;1(6):e0000065. doi: 10.1371/journal.pdig.0000065 36812533 PMC9931316

[5] Chang C-N, Chien H-Y, Malagon-Palacios L. College reopening and community spread of COVID-19 in the United States. Public Health. 2022;204:70–5. doi: 10.1016/j.puhe.2022.01.001 35176623 PMC8747949

[6] Wilson E, Donovan CV, Campbell M, Chai T, Pittman K, Seña AC, et al. Multiple COVID-19 clusters on a university campus - North Carolina, August 2020. MMWR Morb Mortal Wkly Rep. 2020;69:1416–8. 33001871 10.15585/mmwr.mm6939e3 PMC7537562

[7] Quintana C, Stucka M. Astonishingly risky: COVID-19 cases at colleges are fueling the nation’s hottest outbreaks. USA Today. 2020.

[8] Mack K, Yamey G. After cruise ships and nursing homes, will universities be the next COVID-19 tinderboxes? Time. 2020. https://time.com/5867395/will-universities-be-next-covid-19-tinderboxes/

[9] Hubler S, Hartocollis A. How colleges became the new covid hot spots. The New York Times. 2020.

[10] Bahl R, Eikmeier N, Fraser A, Junge M, Keesing F, Nakahata K, et al. Modeling COVID-19 spread in small colleges. PLoS One. 2021;16(8):e0255654. doi: 10.1371/journal.pone.0255654 34407115 PMC8372956

[11] Andersen MS, Bento AI, Basu A, Marsicano CR, Simon KI. College openings in the United States increase mobility and COVID-19 incidence. PLoS One. 2022;17(8):e0272820. doi: 10.1371/journal.pone.0272820 36037207 PMC9423614

[12] Walke HT, Honein MA, Redfield RR. Preventing and Responding to COVID-19 on College Campuses. JAMA. 2020;324(17):1727–8. doi: 10.1001/jama.2020.20027 32991681 PMC9648565

[13] Leidner AJ, Barry V, Bowen VB, Silver R, Musial T, Kang GJ, et al. Opening of Large Institutions of Higher Education and County-Level COVID-19 Incidence - United States, July 6-September 17, 2020. MMWR Morb Mortal Wkly Rep. 2021;70(1):14–9. doi: 10.15585/mmwr.mm7001a4 33411699 PMC7790156

[14] Muller K, Muller PA. Mathematical modelling of the spread of COVID-19 on a university campus. Infect Dis Model. 2021;6:1025–45. doi: 10.1016/j.idm.2021.08.004 34414342 PMC8364150

[15] Poletti P, Tirani M, Cereda D, Trentini F, Guzzetta G, Sabatino G, et al. Association of Age With Likelihood of Developing Symptoms and Critical Disease Among Close Contacts Exposed to Patients With Confirmed SARS-CoV-2 Infection in Italy. JAMA Netw Open. 2021;4(3):e211085. doi: 10.1001/jamanetworkopen.2021.1085 33688964 PMC7948061

[16] Valesano AL, Fitzsimmons WJ, Blair CN, Woods RJ, Gilbert J, Rudnik D, et al. SARS-CoV-2 Genomic Surveillance Reveals Little Spread From a Large University Campus to the Surrounding Community. Open Forum Infect Dis. 2021;8(11):ofab518. doi: 10.1093/ofid/ofab518 34805437 PMC8600169

[17] Park SW, Daskalaki I, Izzo RM, Aranovich I, Te Velthuis AJW, Notterman DA, et al. Relative role of community transmission and campus contagion in driving the spread of SARS-CoV-2: Lessons from Princeton University. PNAS Nexus. 2023;2(7):pgad201. doi: 10.1093/pnasnexus/pgad201 37457892 PMC10338902

[18] Clancey E, Mietchen MS, McMichael C, Lofgren ET. Unexpected Transmission Dynamics in a University Town: Lessons from COVID-19. medRxiv. 2024;:2024.01.10.24301116. doi: 10.1101/2024.01.10.24301116 40838608 PMC12459142

[19] Lu H, Weintz C, Pace J, Indana D, Linka K, Kuhl E. Are college campuses superspreaders? A data-driven modeling study. Comput Methods Biomech Biomed Engin. 2021;24(10):1136–45. doi: 10.1080/10255842.2020.1869221 33439055

[20] Andrews KR, New DD, Gour DS, Francetich K, Minnich SA, Robison BD, et al. Genomic surveillance identifies potential risk factors for SARS-CoV-2 transmission at a mid-sized university in a small rural town. Sci Rep. 2023;13(1):7902. doi: 10.1038/s41598-023-34625-7 37193760 PMC10185956

[21] Tupper P, Colijn C. COVID-19 in schools: Mitigating classroom clusters in the context of variable transmission. PLoS Comput Biol. 2021;17(7):e1009120. doi: 10.1371/journal.pcbi.1009120 34237051 PMC8266060

[22] Lee J, Acosta N, Waddell BJ, Du K, Xiang K, Van Doorn J, et al. Campus node-based wastewater surveillance enables COVID-19 case localization and confirms lower SARS-CoV-2 burden relative to the surrounding community. Water Res. 2023;244:120469. doi: 10.1016/j.watres.2023.120469 37634459

[23] Brown RA. A simple model for control of COVID-19 infections on an urban campus. Proc Natl Acad Sci U S A. 2021;118(36):e2105292118. doi: 10.1073/pnas.2105292118 34475214 PMC8433581

[24] Karmakar M, Lantz PM, Tipirneni R. Association of Social and Demographic Factors With COVID-19 Incidence and Death Rates in the US. JAMA Netw Open. 2021;4(1):e2036462. doi: 10.1001/jamanetworkopen.2020.36462 33512520 PMC7846939

[25] Pijls BG, Jolani S, Atherley A, Derckx RT, Dijkstra JIR, Franssen GHL, et al. Demographic risk factors for COVID-19 infection, severity, ICU admission and death: a meta-analysis of 59 studies. BMJ Open. 2021;11(1):e044640. doi: 10.1136/bmjopen-2020-044640 33431495 PMC7802392

[26] Zhang CH, Schwartz GG. Spatial Disparities in Coronavirus Incidence and Mortality in the United States: An Ecological Analysis as of May 2020. J Rural Health. 2020;36(3):433–45. doi: 10.1111/jrh.12476 32543763 PMC7323165

[27] Doyle-Baker PK, Ladle A, Rout A, Galpern P. Smartphone GPS Locations of Students’ Movements to and from Campus. IJGI. 2021;10(8):517. doi: 10.3390/ijgi10080517

[28] Kirk CM, Lewis RK. Sense of community on an urban, commuter campus. International Journal of Adolescence and Youth. 2013;20(1):48–60. doi: 10.1080/02673843.2013.763833

[29] Bharti N, Lambert B, Exten C, Faust C, Ferrari M, Robinson A. Large university with high COVID-19 incidence is not associated with excess cases in non-student population. Sci Rep. 2022;12(1):3313. doi: 10.1038/s41598-022-07155-x 35228585 PMC8885693

[30] Ciubotariu II, Dorman J, Perry NM, Gorenstein L, Kattoor JJ, Fola AA, et al. Genomic Surveillance of SARS-CoV-2 in a University Community: Insights Into Tracking Variants, Transmission, and Spread of Gamma (P.1) Variant. Open Forum Infect Dis. 2022;9(7):ofac268. doi: 10.1093/ofid/ofac268 35818365 PMC9213861

[31] Dornburg A, Federman S, Lamb AD, Jones CD, Near TJ. Cradles and museums of Antarctic teleost biodiversity. Nat Ecol Evol. 2017;1(9):1379–84. doi: 10.1038/s41559-017-0239-y 29046532

[32] Gilroy JJ, Edwards DP. Source-sink dynamics: A neglected problem for landscape-scale biodiversity conservation in the tropics. Curr Landsc Ecol Rep. 2017;2:51–60.

[33] Goldberg EE, Roy K, Lande R, Jablonski D. Diversity, endemism, and age distributions in macroevolutionary sources and sinks. Am Nat. 2005;165(6):623–33. doi: 10.1086/430012 15937743

[34] Schurr FM, Pagel J, Cabral JS, Groeneveld J, Bykova O, O’Hara BR. How to understand species’ niches and range dynamics: a demographic research agenda for biogeography. Journal of Biogeography. 2012;39:2146–62.

[35] Volz EM, Koelle K, Bedford T. Viral phylodynamics. PLoS Comput Biol. 2013;9(3):e1002947. doi: 10.1371/journal.pcbi.1002947 23555203 PMC3605911

[36] Baele G, Dellicour S, Suchard MA, Lemey P, Vrancken B. Recent advances in computational phylodynamics. Curr Opin Virol. 2018;31:24–32. doi: 10.1016/j.coviro.2018.08.009 30248578

[37] Attwood SW, Hill SC, Aanensen DM, Connor TR, Pybus OG. Phylogenetic and phylodynamic approaches to understanding and combating the early SARS-CoV-2 pandemic. Nat Rev Genet. 2022;23(9):547–62. doi: 10.1038/s41576-022-00483-8 35459859 PMC9028907

[38] Lin SYC, Toyoda H, Kumada T, Liu H-F. Molecular Evolution and Phylodynamics of Acute Hepatitis B Virus in Japan. PLoS One. 2016;11(6):e0157103. doi: 10.1371/journal.pone.0157103 27280441 PMC4900519

[39] Truong Nguyen P, Kant R, Van den Broeck F, Suvanto MT, Alburkat H, Virtanen J, et al. The phylodynamics of SARS-CoV-2 during 2020 in Finland. Commun Med (Lond). 2022;2:65. doi: 10.1038/s43856-022-00130-7 35698660 PMC9187640

[40] Brown P. UNC Charlotte sets historic enrollment record. Inside UNC Charlotte. 2024. https://inside.charlotte.edu/2024/09/05/unc-charlotte-sets-historic-enrollment-record/

[41] Bouckaert R, Vaughan TG, Barido-Sottani J, Duchêne S, Fourment M, Gavryushkina A, et al. BEAST 2.5: An advanced software platform for Bayesian evolutionary analysis. PLoS Comput Biol. 2019;15(4):e1006650. doi: 10.1371/journal.pcbi.1006650 30958812 PMC6472827

[42] Wilmink G, Summer I, Marsyla D, Sukhu S, Grote J, Zobel G, et al. Real-Time Digital Contact Tracing: Development of a System to Control COVID-19 Outbreaks in Nursing Homes and Long-Term Care Facilities. JMIR Public Health Surveill. 2020;6(3):e20828. doi: 10.2196/20828 32745013 PMC7451111

[43] Wen T-H, Hsu C-S, Hu M-C. Evaluating neighborhood structures for modeling intercity diffusion of large-scale dengue epidemics. Int J Health Geogr. 2018;17(1):9. doi: 10.1186/s12942-018-0131-2 29724243 PMC5934834

[44] Soda KJ, Chen X, Feinn R, Hill DR. Monitoring and responding to emerging infectious diseases in a university setting: A case study using COVID-19. PLoS One. 2023;18(5):e0280979. doi: 10.1371/journal.pone.0280979 37196023 PMC10191342

[45] Yaglom HD, Maurer M, Collins B, Hojnacki J, Monroy-Nieto J, Bowers JR, et al. One health genomic surveillance and response to a university-based outbreak of the SARS-CoV-2 Delta AY.25 lineage, Arizona, 2021. PLoS One. 2022;17:e0272830. 10.1371/journal.pone.0272830 PMC9621446 36315517

[46] Kache PA, Santos-Vega M, Stewart-Ibarra AM, Cook EM, Seto KC, Diuk-Wasser MA. Bridging landscape ecology and urban science to respond to the rising threat of mosquito-borne diseases. Nat Ecol Evol. 2022;6(11):1601–16. doi: 10.1038/s41559-022-01876-y 36303000

[47] LaDeau SL, Allan BF, Leisnham PT, Levy MZ. The ecological foundations of transmission potential and vector-borne disease in urban landscapes. Funct Ecol. 2015;29:889–901. doi: 10.1111/1365-2435.12487 26549921 PMC4631442

[48] Hoen AG, Hladish TJ, Eggo RM, Lenczner M, Brownstein JS, Meyers LA. Epidemic Wave Dynamics Attributable to Urban Community Structure: A Theoretical Characterization of Disease Transmission in a Large Network. J Med Internet Res. 2015;17(7):e169. doi: 10.2196/jmir.3720 26156032 PMC4526984

[49] Du Z, Wang L, Shan S, Lam D, Tsang TK, Xiao J, et al. Pandemic fatigue impedes mitigation of COVID-19 in Hong Kong. Proc Natl Acad Sci U S A. 2022;119(48):e2213313119. doi: 10.1073/pnas.2213313119 36417445 PMC9860288

[50] MacIntyre CR, Nguyen P-Y, Chughtai AA, Trent M, Gerber B, Steinhofel K, et al. Mask use, risk-mitigation behaviours and pandemic fatigue during the COVID-19 pandemic in five cities in Australia, the UK and USA: A cross-sectional survey. Int J Infect Dis. 2021;106:199–207. doi: 10.1016/j.ijid.2021.03.056 33771668 PMC7985682

[51] Haktanir A, Can N, Seki T, Kurnaz MF, Dilmaç B. Do we experience pandemic fatigue? current state, predictors, and prevention. Curr Psychol. 2022;41(10):7314–25. doi: 10.1007/s12144-021-02397-w 34690475 PMC8527300

[52] Jalali N, Brustad HK, Frigessi A, MacDonald EA, Meijerink H, Feruglio SL, et al. Increased household transmission and immune escape of the SARS-CoV-2 Omicron compared to Delta variants. Nat Commun. 2022;13(1):5706. doi: 10.1038/s41467-022-33233-9 36175424 PMC9520116

[53] Shrestha LB, Foster C, Rawlinson W, Tedla N, Bull RA. Evolution of the SARS-CoV-2 omicron variants BA.1 to BA.5: Implications for immune escape and transmission. Rev Med Virol. 2022;32(5):e2381. doi: 10.1002/rmv.2381 35856385 PMC9349777

[54] McCallum M, Czudnochowski N, Rosen LE, Zepeda SK, Bowen JE, Walls AC, et al. Structural basis of SARS-CoV-2 Omicron immune evasion and receptor engagement. Science. 2022;375(6583):864–8. doi: 10.1126/science.abn8652 35076256 PMC9427005

[55] Arnold CRK, Srinivasan S, Rodriguez S, Rydzak N, Herzog CM, Gontu A, et al. A longitudinal study of the impact of university student return to campus on the SARS-CoV-2 seroprevalence among the community members. Sci Rep. 2022;12(1):8586. doi: 10.1038/s41598-022-12499-5 35597780 PMC9124192

[56] Yakusheva O, van den Broek-Altenburg E, Brekke G, Atherly A. Lives saved and lost in the first six month of the US COVID-19 pandemic: A retrospective cost-benefit analysis. PLoS One. 2022;17(1):e0261759. doi: 10.1371/journal.pone.0261759 35061722 PMC8782469

[57] Losina E, Leifer V, Millham L, Panella C, Hyle EP, Mohareb AM. College campuses and COVID-19 mitigation: clinical and economic value. Ann Intern Med. 2021;174:472–83. 33347322 10.7326/M20-6558 PMC7755069

[58] Miles D, Stedman M, Heald A. Living with covid-19: balancing costs against benefits in the face of the virus. NIER. 2020;253:R60–76. doi: 10.1017/nie.2020.30

[59] Doidge S, Doyle J. Australian universities in the age of Covid. Educational Philosophy and Theory. 2020;54(6):668–74. doi: 10.1080/00131857.2020.1804343

[60] Ahlburg DA. Covid-19 and UK universities. Polit Q. 2020;91:649–54. 32836411 10.1111/1467-923X.12867 PMC7361847

[61] Ciubotariu II, Wilkes RP, Kattoor JJ, Christian EN, Carpi G, Kitchen A. Investigating the rise of Omicron variant through genomic surveillance of SARS-CoV-2 infections in a highly vaccinated university population. Microb Genom. 2024;10(2):001194. doi: 10.1099/mgen.0.001194 38334271 PMC10926704

[62] Gibas C, Lambirth K, Mittal N, Juel MAI, Barua VB, Roppolo Brazell L, et al. Implementing building-level SARS-CoV-2 wastewater surveillance on a university campus. Sci Total Environ. 2021;782:146749. doi: 10.1016/j.scitotenv.2021.146749 33838367 PMC8007530

[63] Johnson KE, Pasco R, Woody S, Lachmann M, Johnson-Leon M, Bhavnani D, et al. Optimizing COVID-19 testing strategies on college campuses: Evaluation of the health and economic costs. PLoS Comput Biol. 2023;19(12):e1011715. doi: 10.1371/journal.pcbi.1011715 38134223 PMC10773932

[64] Hekmati A, Luhar M, Krishnamachari B, Matarić M. Simulating COVID-19 classroom transmission on a university campus. Proc Natl Acad Sci U S A. 2022;119(22):e2116165119. doi: 10.1073/pnas.2116165119 35609196 PMC9295731

[65] Heesterbeek H, Anderson RM, Andreasen V, Bansal S, De Angelis D, Dye C, et al. Modeling infectious disease dynamics in the complex landscape of global health. Science. 2015;347(6227):aaa4339. doi: 10.1126/science.aaa4339 25766240 PMC4445966

[66] Pybus OG, Rambaut A. Evolutionary analysis of the dynamics of viral infectious disease. Nat Rev Genet. 2009;10(8):540–50. doi: 10.1038/nrg2583 19564871 PMC7097015

[67] Wu Y, Kang L, Guo Z, Liu J, Liu M, Liang W. Incubation Period of COVID-19 Caused by Unique SARS-CoV-2 Strains: A Systematic Review and Meta-analysis. JAMA Netw Open. 2022;5(8):e2228008. doi: 10.1001/jamanetworkopen.2022.28008 35994285 PMC9396366

[68] Jiang X, Rayner S, Luo M-H. Does SARS-CoV-2 has a longer incubation period than SARS and MERS?. J Med Virol. 2020;92(5):476–8. doi: 10.1002/jmv.25708 32056235 PMC7166592

[69] Bergeri I, Whelan MG, Ware H, Subissi L, Nardone A, Lewis HC, et al. Global SARS-CoV-2 seroprevalence from January 2020 to April 2022: A systematic review and meta-analysis of standardized population-based studies. PLoS Med. 2022;19(11):e1004107. doi: 10.1371/journal.pmed.1004107 36355774 PMC9648705

[70] Townsend JP, Hassler HB, Lamb AD, Sah P, Alvarez Nishio A, Nguyen C. Seasonality of endemic COVID-19. mBio. 2023;14:e0142623. 10.1128/mbio.01426-23 PMC10746271 37937979

[71] Townsend JP, Hassler HB, Dornburg A. Optimal Annual COVID-19 Vaccine Boosting Dates Following Previous Booster Vaccination or Breakthrough Infection. Clin Infect Dis. 2025;80(2):316–22. doi: 10.1093/cid/ciae559 39589144 PMC11848277

[72] Bansal S, Chowell G, Simonsen L, Vespignani A, Viboud C. Big Data for Infectious Disease Surveillance and Modeling. J Infect Dis. 2016;214(suppl_4):S375–9. doi: 10.1093/infdis/jiw400 28830113 PMC5181547

[73] Russell KL, Broderick MP, Franklin SE, Blyn LB, Freed NE, Moradi E, et al. Transmission dynamics and prospective environmental sampling of adenovirus in a military recruit setting. J Infect Dis. 2006;194(7):877–85. doi: 10.1086/507426 16960774 PMC7109706

[74] Gardner W, States D, Bagley N. The Coronavirus and the Risks to the Elderly in Long-Term Care. J Aging Soc Policy. 2020;32(4–5):310–5. doi: 10.1080/08959420.2020.1750543 32245346

[75] Dykgraaf SH, Matenge S, Desborough J, Sturgiss E, Dut G, Roberts L, et al. Protecting Nursing Homes and Long-Term Care Facilities From COVID-19: A Rapid Review of International Evidence. J Am Med Dir Assoc. 2021;22(10):1969–88. doi: 10.1016/j.jamda.2021.07.027 34428466 PMC8328566

[76] Pater AA, Bosmeny MS, White AA, Sylvain RJ, Eddington SB, Parasrampuria M, et al. High throughput nanopore sequencing of SARS-CoV-2 viral genomes from patient samples. J Biol Methods. 2021;8(COVID 19 Spec Iss):e155. doi: 10.14440/jbm.2021.360 34631911 PMC8493558

[77] Ferdous J, Weathers T, Bharati Barua V, Stiers E, France A, C Lambirth K, et al. A SARS-CoV-2 Surveillance Sequencing Protocol Optimized for Oxford Nanopore PromethION v1. Springer Science and Business Media LLC. 2021. doi: 10.17504/protocols.io.butbnwin

[78] Aksamentov I, Roemer C, Hodcroft E, Neher R. Nextclade: clade assignment, mutation calling and quality control for viral genomes. JOSS. 2021;6(67):3773. doi: 10.21105/joss.03773

[79] Katoh K, Standley DM. MAFFT multiple sequence alignment software version 7: improvements in performance and usability. Mol Biol Evol. 2013;30(4):772–80. doi: 10.1093/molbev/mst010 23329690 PMC3603318

[80] De Maio N, Kalaghatgi P, Turakhia Y, Corbett-Detig R, Minh BQ, Goldman N. Maximum likelihood pandemic-scale phylogenetics. Nat Genet. 2023;55(5):746–52. doi: 10.1038/s41588-023-01368-0 37038003 PMC10181937

[81] Miura S, Tamura K, Tao Q, Huuki LA, Kosakovsky Pond SL, Priest J, et al. A new method for inferring timetrees from temporally sampled molecular sequences. PLoS Comput Biol. 2020;16(1):e1007046. doi: 10.1371/journal.pcbi.1007046 31951607 PMC7018096

[82] Kumar S, Stecher G, Li M, Knyaz C, Tamura K. MEGA X: Molecular Evolutionary Genetics Analysis across Computing Platforms. Mol Biol Evol. 2018;35(6):1547–9. doi: 10.1093/molbev/msy096 29722887 PMC5967553

[83] Tamura K, Tao Q, Kumar S. Theoretical Foundation of the RelTime Method for Estimating Divergence Times from Variable Evolutionary Rates. Mol Biol Evol. 2018;35(7):1770–82. doi: 10.1093/molbev/msy044 29893954 PMC5995221

[84] Tamura K, Battistuzzi FU, Billing-Ross P, Murillo O, Filipski A, Kumar S. Estimating divergence times in large molecular phylogenies. Proc Natl Acad Sci U S A. 2012;109(47):19333–8. doi: 10.1073/pnas.1213199109 23129628 PMC3511068

[85] Mello B, Tao Q, Tamura K, Kumar S. Fast and Accurate Estimates of Divergence Times from Big Data. Mol Biol Evol. 2017;34(1):45–50. doi: 10.1093/molbev/msw247 27836983

[86] Townsend JP, Hassler HB, Wang Z, Miura S, Singh J, Kumar S, et al. The durability of immunity against reinfection by SARS-CoV-2: a comparative evolutionary study. Lancet Microbe. 2021;2(12):e666–75. doi: 10.1016/S2666-5247(21)00219-6 34632431 PMC8486316

[87] Drummond AJ, Ho SYW, Phillips MJ, Rambaut A. Relaxed phylogenetics and dating with confidence. PLoS Biol. 2006;4(5):e88. doi: 10.1371/journal.pbio.0040088 16683862 PMC1395354

[88] Lemey P, Rambaut A, Drummond AJ, Suchard MA. Bayesian phylogeography finds its roots. PLoS Comput Biol. 2009;5(9):e1000520. doi: 10.1371/journal.pcbi.1000520 19779555 PMC2740835

[89] Townsend JP, Hassler HB, Sah P, Galvani AP, Dornburg A. The durability of natural infection and vaccine-induced immunity against future infection by SARS-CoV-2. Proc Natl Acad Sci U S A. 2022;119(31):e2204336119. doi: 10.1073/pnas.2204336119 35858382 PMC9351502

[90] Pagel M, Meade A, Barker D. Bayesian estimation of ancestral character states on phylogenies. Syst Biol. 2004;53(5):673–84. doi: 10.1080/10635150490522232 15545248

[91] Chazot N, Condamine FL, Dudas G, Peña C, Kodandaramaiah U, Matos-Maraví P, et al. Conserved ancestral tropical niche but different continental histories explain the latitudinal diversity gradient in brush-footed butterflies. Nat Commun. 2021;12(1):5717. doi: 10.1038/s41467-021-25906-8 34588433 PMC8481491

[92] Cincotta A, Nicolaï M, Campos HBN, McNamara M, D’Alba L, Shawkey MD, et al. Pterosaur melanosomes support signalling functions for early feathers. Nature. 2022;604(7907):684–8. doi: 10.1038/s41586-022-04622-3 35444275 PMC9046085

[93] Lemey P, Rambaut A, Welch JJ, Suchard MA. Phylogeography takes a relaxed random walk in continuous space and time. Mol Biol Evol. 2010;27(8):1877–85. doi: 10.1093/molbev/msq067 20203288 PMC2915639

[94] Rambaut A, Drummond AJ, Xie D, Baele G, Suchard MA. Posterior Summarization in Bayesian Phylogenetics Using Tracer 1.7. Syst Biol. 2018;67(5):901–4. doi: 10.1093/sysbio/syy032 29718447 PMC6101584

[95] Gu Z, Gu L, Eils R, Schlesner M, Brors B. Circlize Implements and enhances circular visualization in R. Bioinformatics. 2014;30(19):2811–2. doi: 10.1093/bioinformatics/btu393 24930139

[96] Wickham H. Ggplot2. Wiley Interdiscip Rev Comput Stat. 2011;3:180–5.

[97] Yu G. Data integration, manipulation and visualization of phylogenetic trees. Boca Raton: Chapman and Hall/CRC. 2022.

[98] Wang L-G, Lam TT-Y, Xu S, Dai Z, Zhou L, Feng T, et al. Treeio: An R Package for Phylogenetic Tree Input and Output with Richly Annotated and Associated Data. Mol Biol Evol. 2020;37(2):599–603. doi: 10.1093/molbev/msz240 31633786 PMC6993851

[99] Wickham H, Averick M, Bryan J, Chang W, McGowan L, François R, et al. Welcome to the Tidyverse. JOSS. 2019;4(43):1686. doi: 10.21105/joss.01686

