# Supplementary figures and images for: On campus dormitories as viral transmission sinks: Phylodynamic insights into student housing networks during the COVID-19 pandemic
Source: PLoS Pathog. 2025 Nov 3;21(11):e1013666. doi: 10.1371/journal.ppat.1013666 (PMC12594326; doi:10.1371/journal.ppat.1013666)

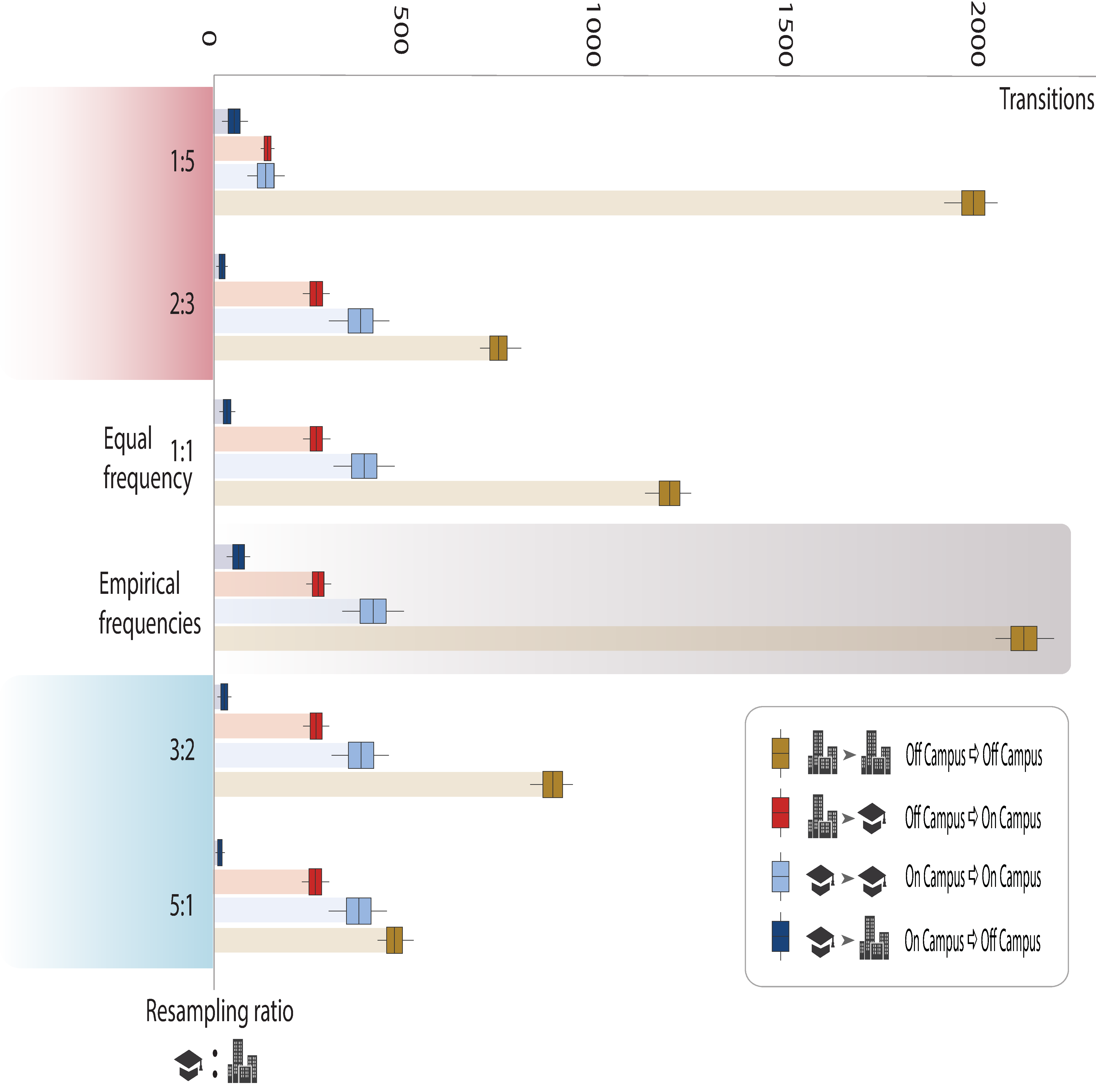

Supplement: S1 Fig — State transition frequencies were evaluated across phylogenies randomly subsampled at predefined on-campus:off-campus sampling ratios (1:5, 2:3, 1:1, 3:2, 5:1). Transition categories (off-campus to off-campus, on-campus to on-campus, off-campus to on-campus, and on-campus to off-campus) were compared across biased sampling strategies to assess if uneven frequencies of infection between populations would impact the trends observed in the empirical frequencies. Across all sampling strategies, the relative relationship between transition frequencies remained consistent, with only the absolute number of transmission events within the off-campus community modulating. Bars represent the mean number of transitions across each simulation and category, with box plots indicating the 95% confidence interval for each distribution. The x-axis denotes the resampling ratio, and the y-axis indicates the number of inferred transmission events. Off campus and on campus biased resampling results are indicated by shading on the x-axis. (TIF) [file ppat.1013666.s001.tif]

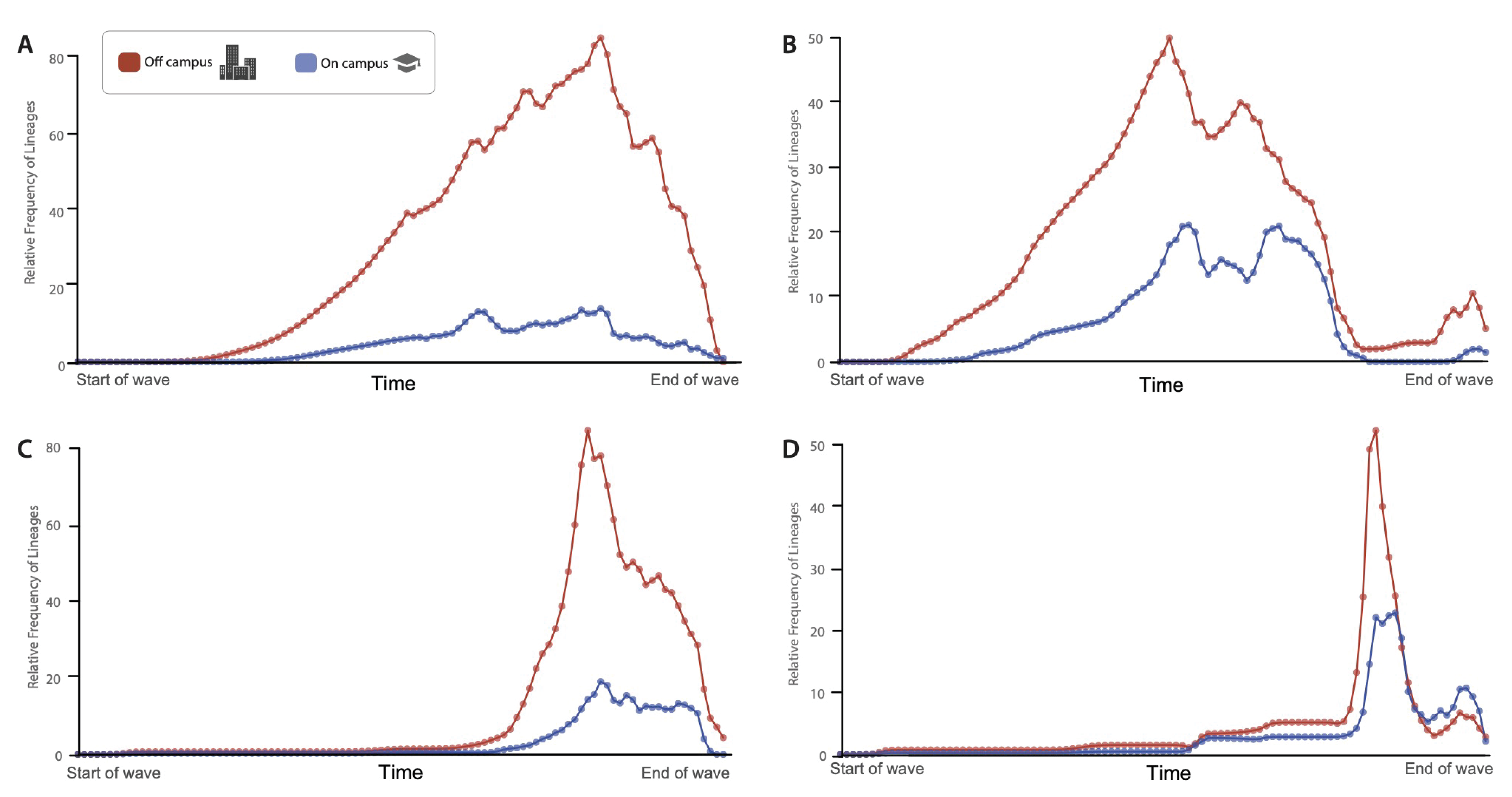

Supplement: S2 Fig — Lines indicate the relative frequency of SARS-COV-2 lineages through time for off-campus (warm shading) and on-campus (cool shading). Circles in lineage through time plots represent specific time points. (TIF) [file ppat.1013666.s002.tif]

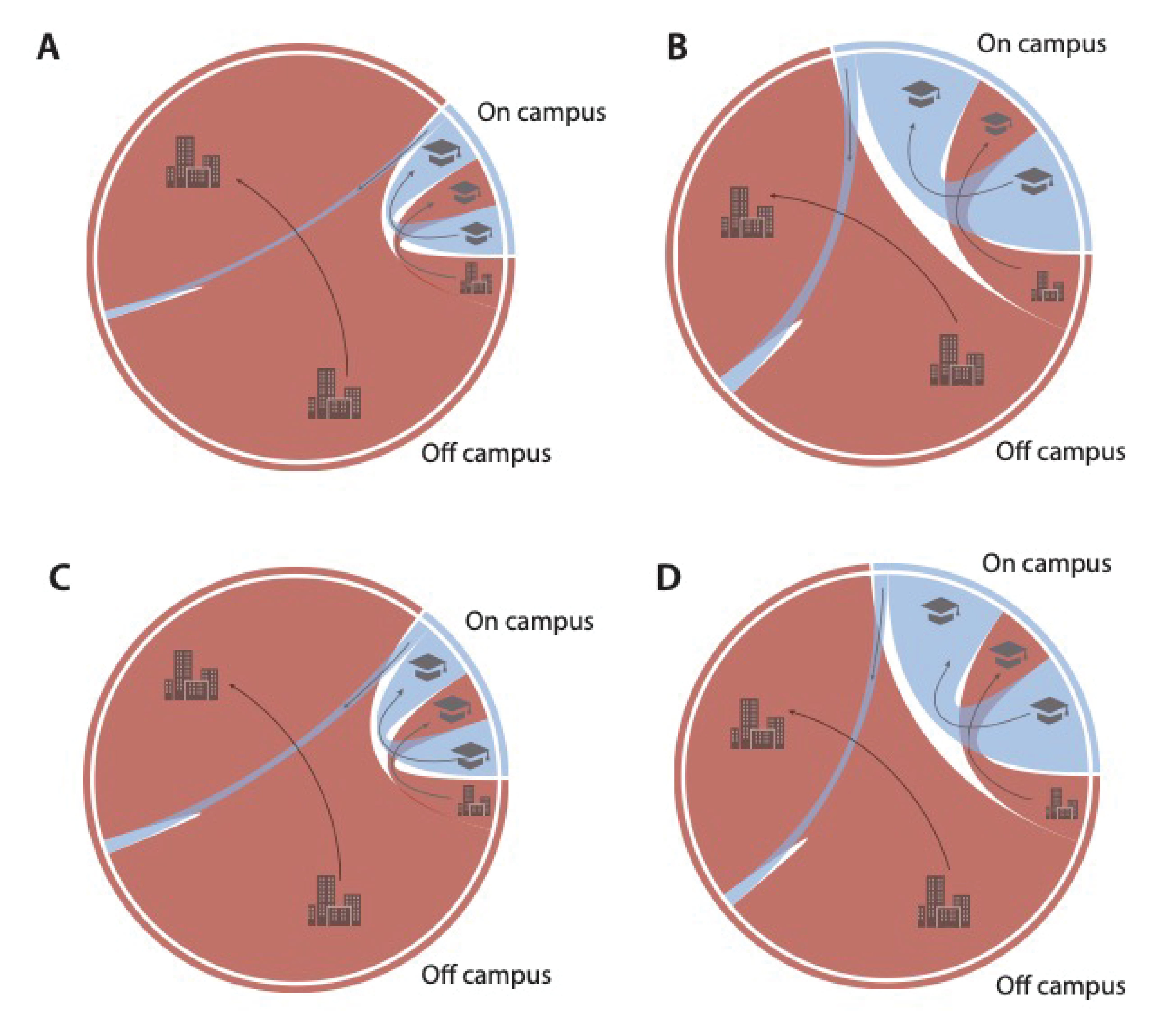

Supplement: S3 Fig — The relative frequency of transmissions between and within communities is depicted in chord diagram, shaded by community. Outer bands correspond to total relative cases for each community, and inner chords illustrate transmission mode scaled to their frequency. Arrows and cartoons illustrate the direction of transmission. These visualizations complement the data presented in S1 Table and illustrate the consistent asymmetry of transmission from off-campus to on-campus populations across all pandemic phases. (TIF) [file ppat.1013666.s003.tif]
