# Supplementary material for: On campus dormitories as viral transmission sinks: Phylodynamic insights into student housing networks during the COVID-19 pandemic
Source: PLoS Pathog. 2025 Nov 3;21(11):e1013666. doi: 10.1371/journal.ppat.1013666 (PMC12594326; doi:10.1371/journal.ppat.1013666)
Supplement: S1 Table — (DOCX) [file ppat.1013666.s004.docx]

| **S1 Table. Mean number of transmissions estimated within and between populations during major waves of the COVID-19 Pandemic** | | | |
| --- | --- | --- | --- |
| **Wave** | **From** | **To** | **Transitions** |
| **Wuhan** | Off Campus | Off Campus | 500 |
|  | Off Campus | On Campus | 48 |
|  | On Campus | Off Campus | 8 |
|  | On Campus | On Campus | 48 |
| **Alpha** | Off Campus | Off Campus | 305 |
|  | Off Campus | On Campus | 58 |
|  | On Campus | Off Campus | 15 |
|  | On Campus | On Campus | 98 |
| **Delta** | Off Campus | Off Campus | 644 |
|  | Off Campus | On Campus | 64 |
|  | On Campus | Off Campus | 17 |
|  | On Campus | On Campus | 74 |
| **Omicron** | Off Campus | Off Campus | 658 |
|  | Off Campus | On Campus | 102 |
|  | On Campus | Off Campus | 21 |
|  | On Campus | On Campus | 190 |
