# Supplementary material for: On campus dormitories as viral transmission sinks: Phylodynamic insights into student housing networks during the COVID-19 pandemic
Source: PLoS Pathog. 2025 Nov 3;21(11):e1013666. doi: 10.1371/journal.ppat.1013666 (PMC12594326; doi:10.1371/journal.ppat.1013666)
Supplement: S2 Table — Variant phases were defined based on sampling dates aligned with periods of variant dominance Wuhan (Fall 2020), Alpha (Spring–Summer 2021), Delta (Fall 2021), and Omicron (Spring 2022). Counts reflect genomes passing quality control filters. The Wuhan reference genome used for rooting the phylogeny is excluded from these totals. (DOCX) [file ppat.1013666.s005.docx]

**S2 Table. Number of high quality SARS-CoV-2 genomes sequenced per variant phase. Variant phases were defined based on sampling dates aligned with periods of variant dominance Wuhan (Fall 2020), Alpha (Spring–Summer 2021), Delta (Fall 2021), and Omicron (Spring 2022). Counts reflect genomes passing quality control filters. The Wuhan reference genome used for rooting the phylogeny is excluded from these totals.**

| **Year** | **Pandemic Phase** | **Building Group** | **Genomes Sequenced** |
| --- | --- | --- | --- |
| **2022** | Omicron | Off Campus | 300 |
|  |  | On Campus | 187 |
| **2021** | Delta | Off Campus | 292 |
|  |  | On Campus | 109 |
|  | Alpha | Off Campus | 132 |
|  |  | On Campus | 107 |
| **2020** | Wuhan | Off Campus | 234 |
|  |  | On Campus | 69 |
