# Supplementary material for: On campus dormitories as viral transmission sinks: Phylodynamic insights into student housing networks during the COVID-19 pandemic
Source: PLoS Pathog. 2025 Nov 3;21(11):e1013666. doi: 10.1371/journal.ppat.1013666 (PMC12594326; doi:10.1371/journal.ppat.1013666)
Supplement: S1 Text — (DOCX) [file ppat.1013666.s006.docx]

**Supplemental Materials For:**

**Viral Dispersal Patterns in University Communities: analysis of comprehensive COVID-19 sequencing on an urban university campus.**

Juan Bolanos, Alex Dornburg, April Harris, Samuel Kunkleman, Jannatul Ferdous Moon, William Taylor, Jessica Schlueter, Cynthia Gibas

**Supplemental Results**

*COVID-19 intervention strategies shifted through time at UNCC*

Throughout the study period, the University of North Carolina at Charlotte (UNCC) implemented a multi-layered mitigation strategy, including thrice-weekly wastewater surveillance at the building and neighborhood levels, with follow-up clinical testing for positive wastewater signals [[1]](https://paperpile.com/c/vrgl0l/g1OK). Following the onset of the COVID-19 pandemic, UNCC resumed in-person operations with reduced occupancy in on-campus housing in September 2020. Throughout the 2020-21 academic year, dormitory populations were reduced to one-third of typical capacity, masking and social distancing were required, and students testing positive for SARS-CoV-2 were relocated to a designated isolation dormitory. Additional measures implemented during this included symptomatic testing, mandatory asymptomatic testing before the start of each semester, an in-house contact tracing program, and voluntary self-reporting of symptoms through a daily survey. These extensive efforts earned UNCC the AASCU Excellence and Innovation Award for Campus Pandemic Response [[2]](https://paperpile.com/c/vrgl0l/FnfZ). However, campus COVID-19 mitigation strategies evolved in response to shifting pandemic conditions (**Figure 1**). Over the course of the 2020-2021 academic year, mitigation measures were gradually relaxed, and adherence to daily symptom reporting via health surveys steadily declined. By August of 2021, dormitories were reopened at full capacity. Following the return to campus, the emergence of the delta variant catalyzed a reinstatement of prior mitigation strategies, including masking and social distancing strategies as well as building-wide testing after detection of virus in building wastewater and increased re-entry and random asymptomatic testing. This was combined with incentivization of vaccine adoption, by allowing exemption from routine individual testing. Throughout the course of the fall semester, these measures again relaxed. By the time of the peak omicron wave in January 2022, in-person learning resumed with significantly reduced mitigation protocols, rendering the majority of previous strategies optional. This shift marked a transition from structured, enforced measures to a reliance on personal responsibility, mirroring broader trends in institutional pandemic responses reflecting changing public health guidance [[3]](https://paperpile.com/c/vrgl0l/pCXN) as well as shifts in the immune status of the general population following infection or vaccination [[4,5]](https://paperpile.com/c/vrgl0l/9QlY+zWbZ).

**References**

1. [Gibas C, Lambirth K, Mittal N, Juel MAI, Barua VB, Roppolo Brazell L, et al. Implementing building-level SARS-CoV-2 wastewater surveillance on a university campus. Sci Total Environ. 2021;782: 146749.](http://paperpile.com/b/vrgl0l/g1OK)

2. [CStandard. It’s Official: UNC Charlotte's Pandemic Response Is Excellent And Innovative. In: Collegiate Standard [Internet]. 21 Aug 2021 [cited 31 Jan 2025]. Available:](http://paperpile.com/b/vrgl0l/FnfZ) <https://collegiatestandard.com/its-official-unc-charlottes-pandemic-response-is-excellent-and-innovative/>

3. [Tomori C, Ahmed A, Evans DP, Meier BM, Nair A. Your health is in your hands? US CDC COVID-19 mask guidance reveals the moral foundations of public health. EClinicalMedicine. 2021;38: 101071.](http://paperpile.com/b/vrgl0l/pCXN)

4. [Townsend JP, Hassler HB, Sah P, Galvani AP, Dornburg A. The durability of natural infection and vaccine-induced immunity against future infection by SARS-CoV-2. Proc Natl Acad Sci U S A. 2022;119: e2204336119.](http://paperpile.com/b/vrgl0l/9QlY)

5. [Townsend JP, Hassler HB, Wang Z, Miura S, Singh J, Kumar S, et al. The durability of immunity against reinfection by SARS-CoV-2: a comparative evolutionary study. Lancet Microbe. 2021;2: e666–e675.](http://paperpile.com/b/vrgl0l/zWbZ)
